# Comparability of in-person and web screening: Does mode affect what households report?

**DOI:** 10.1371/journal.pone.0277017

**Published:** 2022-10-31

**Authors:** Jessica E. Graber, Douglas Williams, Jason Clark

**Affiliations:** 1 National Center for Health Statistics, Hyattsville, Maryland, United States of America; 2 Bureau of Labor Statistics, Washington, District of Columbia, United States of America; 3 Westat, Rockville, Maryland, United States of America; Charles Sturt University - Port Macquarie Campus, AUSTRALIA

## Abstract

Household screening is common when information about characteristics of household members is needed for selection of survey respondents. When key characteristics have a low prevalence, or are oversampled, this can result in a large number of sampled households screened, many of which have no persons selected. For in-person surveys this can be inefficient and costly, especially in an environment of declining response. A multimode design using a mail, push-to-web approach is an attractive alternative due to lower cost and high internet penetration. However, little is known about the comparable data quality properties between in-person and web modes. While in-person screening is considered a gold standard approach, respondents may fail to report household members and interviewers may unintentionally screen out reluctant respondents. Similarly, those self-responding sometimes fail to report unrelated household members or young children. In this study we compared in-person and web screening in the National Health and Nutrition Examination Survey. Households were randomly selected to complete a self-administered web screener and subsequently be screened by an interviewer during an in-person visit. We report on the comparability of household characteristics between modes to determine if web screening provides data equivalent to in-person screening. We examine time between the web and in-person screening to see if true change can account for differences. In the presence of conflicting data, we examine selection criteria based on the screening responses to see how inaccuracies affect selection status, or if inaccuracies or person omissions are systematically related to a specific mode. Approximately 93% (80/86) of households agreed on selection status between the web and in-person modes. Household composition matched fully for 84% (72/86) of households. These results indicate that web screening is a viable option enumerating households in population surveys.

## Introduction

Household screening is a common practice when surveys seek to identify populations with a specific characteristic, or when sampling protocols target specific subgroups. The screening process can vary from general questions asking about the presence of a characteristic (e.g., presence of children, or chronic health condition) to detailed questions about each household member, resulting in a household roster. Studies that sample from a roster of all (or adult) members within each household are frequently conducted using in-person data collection and associated with burdensome or complex questionnaires. The in-person mode aids in motivating response [1], especially when the screening stage is the household’s first introduction to the study and information about who lives at the sampled unit can be viewed as intrusive [2–5].

When surveys target, or oversample, specific population subgroups, the proportion of sampled households where a resident is selected can be low. This can be costly due to the high number of households that require an in-person visit, but yield no household members selected for the survey after screening. For example, the National Survey of Family Growth (NSFG) targets persons aged 15 to 49 with oversampling of different groups based on race and ethnicity [6], with one age-eligible person selected at every household containing an age-eligible member. This resulted in only 58 percent of all screened households with a resident selected. Another survey, the Health and Retirement Study, targets adults in the U.S. over age 50, with new cohorts added every six years [7]. Based on age eligibility and race and ethnicity oversampling, only about 18 percent of screened households in the 2010 sample supplement included a household member selected for the study [8].

These surveys show the effect of target criteria and oversampling on sample yield, but the same low yields can occur when sampling criteria rely on multiple factors. From 1999 to 2020, the National Health and Nutrition Examination Survey (NHANES) targeted all civilian, noninstitutionalized persons in the U.S., but sampled persons from residential households based on various combinations of age, gender, race and ethnicity, (and in select cases income) with oversampling for several race and ethnicity groups [9]. To obtain the desired distributions in the sample, many screened households may not have had any members selected. In the 2017–2018 data collection cycle, this resulted in only 35 percent of all screened households having a resident selected to participate in the survey. These studies show that considerable effort is expended in the process of identifying target population members, or identifying household members for the purpose of sampling from under-represented groups.

One approach to mitigating the cost and effort of in-person data collection is to adopt a sequential mixed-mode approach, incorporating self-administered data collection [10–12]. For surveys that screen large numbers of households, this has the potential to yield dramatic savings when large proportions of households are expected to have all household members be ineligible or not selected. For in-person surveys, this can eliminate the need to visit many households. For example, Wagner and colleagues [8] tested a mixed-mode approach on the Health and Retirement Survey, finding positive effects on response, but noting that the sequence of modes may play an important role, mitigating some cost benefits.

A concern with mixing data collection modes is the potential for mode effects. Much research is available comparing interviewer-administered and self-administered modes [13–17]. However, these investigations focus on issues of coverage, nonresponse, or data quality for reported behaviors, activities, or attitudes. Little research is available directly comparing household compositions collected by interviewer-administered and self-administered modes, yet both have known biases for some key groups. What is critical to know is whether these biases are compounded, resulting in negative influences on eligibility or selection rates.

For surveys that enumerate households using a self-administered mode, one primary issue is literacy. This can lead to the complete omission of a household or household members [18], or affect the ability to understand residency questions or instructions [19]. When literacy is not an issue, the complex nature of some households make instructions for whom to include difficult or lacking in logic and frequently not read, leading to erroneous omissions [20,21]. Additionally, systematic biases exist, with self-administered surveys frequently under-reporting children or young babies [22,23].

Enumerating households using an interviewer-administered, in-person mode, can address issues of respondent literacy, or the interviewer can help navigate complex household structures. However, the in-person mode remains subject to both respondent and interviewer influence. Household members with tenuous connections to others within the household continue to be missed [24]. Tourangeau, Kreuter, and Eckman [25] observed both respondent and interviewer influences on roster omissions. They note that when survey eligibility criteria are clear, respondents may exclude household members, or fail to participate entirely. Interviewer influences were noted for productive interviewers who may have screened out eligible households that were uncooperative so that high screener response rates are maintained.

Mode differences in the characteristics of household members for a sampled dwelling unit (DU) can be problematic if they lead to different selection outcomes. If persons in the household are not reported when a household respondent completes a web screener, this can lead to undercoverage and decrease sampling efficiency as more households are needed to meet selection criteria. If some characteristics are not provided, for example, age, race and ethnicity, or income, enough information to determine eligibility or selection status may not be provided, requiring a potentially unnecessary personal visit to a DU.

Clearly, enumerating households for the purpose of identifying or selecting persons is challenging, with mode one of several possible sources of error. In-person screening is generally considered a superior approach, due to higher rates of response and the availability of an interviewer to navigate complexities. However, in-person interviews are the most costly to conduct, so in the era of declining response for in-person surveys [26,27], mixed-mode approaches are increasingly important. Further, survey organizations are trying to reduce unnecessary in-person contacts that may not be welcomed by respondents who have concerns about personal contact.

In this paper, we report on a pilot test comparing information about household members collected by web and in-person modes. The purpose was to determine the feasibility of adding a self-administered web mode for screening households by first determining equivalence to screening by an in-person mode. We conducted a within-subjects design and report on household level consistency between modes and any differential effects on selection based on reported household member characteristics. Consistency in reported person level characteristics between modes are presented along with key patterns in discrepancies.

## Methods

NHANES is a national survey of the civilian, noninstitutionalized U.S. population conducted annually across the U.S. by the National Center for Health Statistics (NCHS). The 2017–2018 NHANES data collection cycle was conducted in 30 primary sampling units (PSUs). In-person interviewing was conducted in each PSU for 10 weeks and included three components. All sampled DUs were first screened to determine if any household member would be selected to participate. Age, gender, race and ethnicity, and income were used as selection criteria for oversampling of specific subgroups. Selected persons (SPs) identified during screening were then asked to complete an interview in their home. Finally, SPs were asked to complete a medical examination at a Mobile Examination Center administered by trained personnel. During the 2017–2018 data collection cycle, 23,722 eligible DUs were identified, and screening information was obtained from 21,559 (90.9 percent). From those responding DUs, 16,211 eligible individuals were selected, 9,254 participated in an interview, and 8,704 were examined (for an overall examination response rate of 48.8 percent). Additional information about the NHANES survey design, response rates, and data collection methodology is available at https://wwwn.cdc.gov/nchs/nhanes/default.aspx.

The NCHS Research Ethics Review Board (ERB) reviewed the NHANES survey design and protocol and approved the study. Health information collected in the NHANES is kept in strictest confidence. During the informed consent process, survey participants were assured that data collected would be used only for stated purposes and would not be disclosed or released to others without the consent of the individual or the establishment in accordance with section 308(d) of the Public Health Service Act (42 U.S.C. 242m). Written consent was obtained for all participants, including parents or guardians of participating minor children. Assent from children ages 7–17 was also collected. For cognitively impaired persons, consent was obtained from guardians, parents, or representatives who had the legal right to consent to medical care on behalf of the impaired person.

Our data come from a pilot test of a web screener conducted in 6 PSUs in 2018. The purpose of the pilot was to administer screening in both self-administered web and interviewer-administered in-person modes so that data quality and accuracy could be assessed between the two. There was concern that the additional web screening request would add burden or the request alone would discourage participation in the in-person screening. To mitigate this issue, sampled DUs within each PSU were randomly assigned to one of two screener conditions; one-half of the sample was assigned to in-person only screening, and the other was assigned to both self-administered web screening and then interviewer administered in-person screening (hereinafter referred to as “web screener.”).

DUs assigned the web screener were administered the screener questionnaire during their in-person visit, regardless of whether any household members were selected by the original self-administered web submission. Field interviewers were not informed about which cases were assigned to each condition nor were they provided with data collected from the web submissions. Interviewers administered the in-person screener uniformly across experimental groups. Across all PSUs a total of 2,107 DUs were assigned to the web screener.

**Fig 1:**
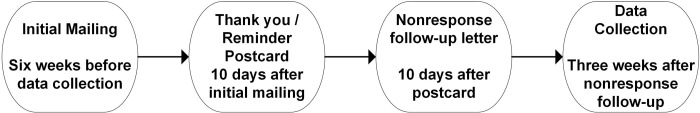
Mail contact protocol and timing for web screening.

The series of contacts and contact timing for the web screener are shown in Fig 1. Sampled DUs were invited to complete the web screener by mail starting six weeks before each PSU opened. This limited our mailing protocol to three contacts. The mailing schedule included an initial invitation letter, a reminder postcard, and a final nonresponse follow-up letter. No monetary incentive was included with the mailed requests to complete the web screener, since DUs completing the in-person screener currently do not receive such incentives. All contact materials included the URL for the web screening questionnaire and a unique username and password. After the initial invitation letter, the reminder postcard was mailed about 10 days later, and the final nonresponse follow-up letter was mailed about 10 days after the postcard. This placed the final contact to complete the web screener about three weeks before field interviewers began in-person contacts.

## Data and analyses

We assessed the quality of the household information collected with the web screener by comparing it to what was collected during the subsequent in-person interview. While information collected from interviewer administered screening is subject to misreporting [25], interviewer administration has been relied upon since the beginning of the current NHANES continuous design starting in 1999. Therefore, we use the in-person data as our standard for comparison.

We are comparing households where one or more household members was selected as eligible for NHANES to those where no household members were selected. In 2018, less than 40 percent of all sampled DUs yielded any eligible household members. We classify households as a complete match if characteristics of all household members match between the in-person and web screeners; a partial match if one or more persons, but not all match (or there are missing or surplus household members); or not a match, if no member of the household matches between modes.

If web screening was fully adopted, identifying households with no SPs using a web screener would remove them from any in-person field activity. Some error, or person missingness, in household characteristics is acceptable in a web screener if the eligibility or selection status of all members of the household remains the same. This is because households where the web screener indicates any members are selected will be visited to complete the interview, and household information can be verified and corrected as needed in these households. For our pilot test, we assess the agreement in determining selection status for all household members between the web and in-person modes by calculating a Kappa statistic when comparing the two modes.

We include an examination of the length of time between the web and in-person screener completion to determine whether the amount of time between these affects non-matching statuses. Non-matching DUs and partial matches may be caused by true changes in household composition between the two modes. We then examine data quality which includes the completeness of reported characteristics and any differences in matching quality between characteristics. This is done by examining person pairs, which we define as the sum of unique persons identified in both the web and the in-person screener that we have determined to be the same person. We conclude our examination by looking at the match between modes for personal identifiers and observable characteristics. Unique, personal identifiers are important, especially in cases where there is more than one household member with matching characteristics, and include first and last name. Observable characteristics included: gender, age, race, and ethnicity.

## Results

The web screener was completed by a small number of sampled DUs. A total of 96 web screeners were completed, yielding an American Association for Public Opinion Research (AAPOR) RR1 [28] of 4.7 percent.

### Match quality between web and in-person

In-person screening interviews were completed with 86 of the 96 (90%) sampled DUs completing a web screener. Table 1 shows the match detail across DUs between the web and in-person screener for the DUs that completed the web screener. In Table 1, Partial Match Web+ indicates an additional person(s) was listed in the web mode, while Partial Match In-person+ indicates an additional person(s) was listed for the in-person mode. A status of No Match indicates that the person or persons listed do not match and indicate completely different households. Finally, No In-person Screener indicates that the screener was not completed for the in-person mode.

**Table 1:** Distribution of match results between web and in-person modes.

Status	Count	Percentage among SPs completing the In-Person Screener
Complete Match	72	83.7%
Partial Match Web+^a^	3	3.5%
Partial Match In-Person+^a^	8	9.3%
No Match	3	3.5%
No In-Person Screener	10	
Total dwelling units	96	

^a^ Partial Match Web+ indicates an additional person(s) was listed in the web mode; Partial Match In-Person+ indicates an additional person(s) was listed in the in-person mode.

The results in Table 1 show that for 84 percent of the DUs completing both the web and in-person screener the household composition matched completely. Another 13 percent matched at least one person, but additional household members were reported in one of the modes, usually for the in-person mode. Finally, 3 percent of the DUs did not match at all.

**Fig 2:**
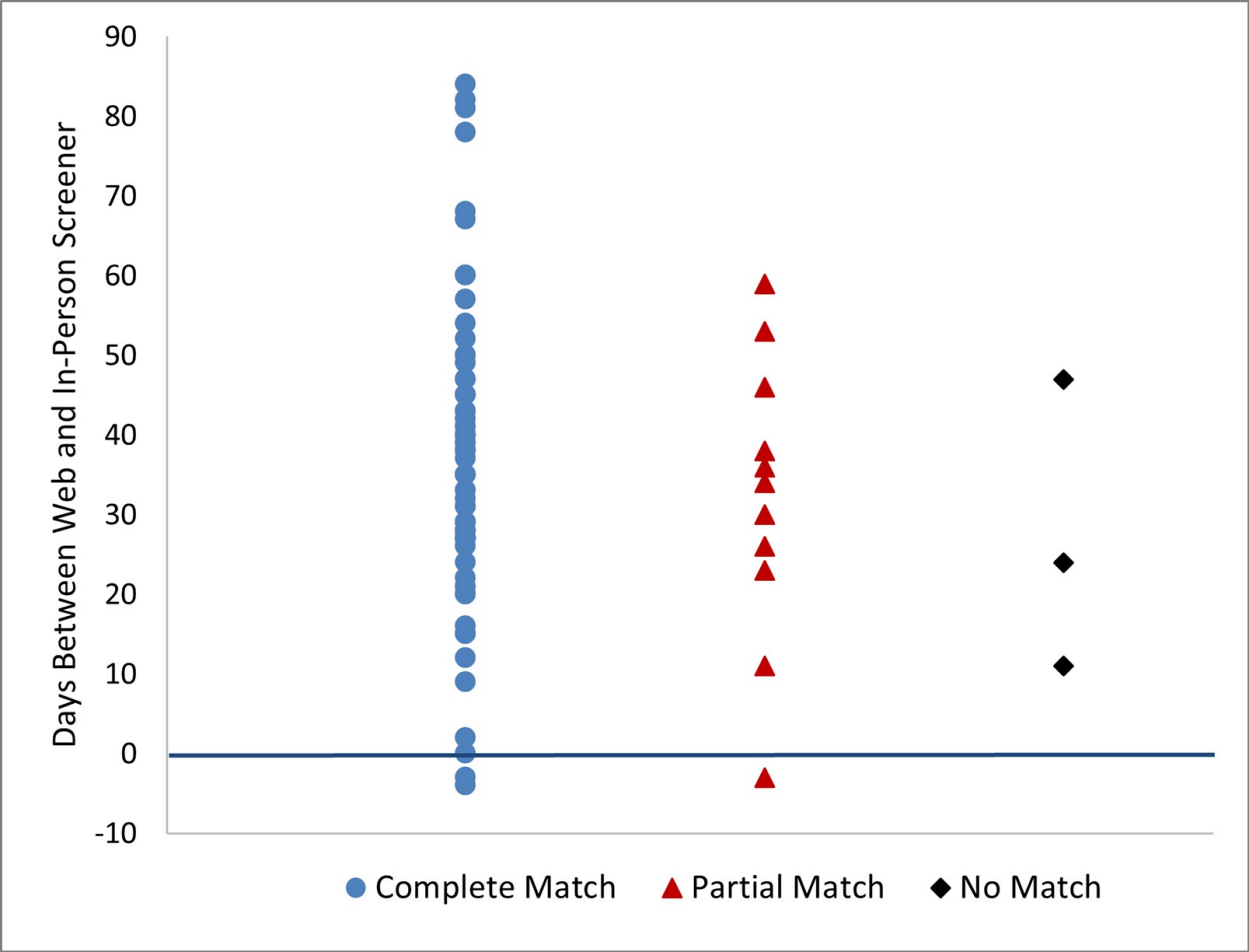
Distribution of time between web screener and in-person screener completion.

The time between web and in-person screener completion is plotted in Fig 2 by matching status. Due to operational issues such as interviewer workload and time needed to make contact with a household, up to 12 weeks could pass between the web and in-person screener completion. Cases plotted with a zero or negative value indicate that the web screener was completed after the in-person screener. A review of these cases suggest that a household member attempted to correct information previously provided during an in-person visit. The partial and no match statuses did not occur more often as time increased. The average time between modes was 36 days for complete matches and 32 days for all others (combining partial and no match groups).

### Accuracy of eligibility status between web and in-person

Table 2 shows that the agreement between the web and in-person screeners was high in determining whether any household members were selected, with 93 percent of cases selected by web screening showing agreement with in-person screening. The Kappa value for the comparison yielded a value of 0.84 conforming to very good agreement [29]. Disagreement was low and nearly equivalent in direction.

**Table 2:** Comparison of web screener selection status with in-person.

		In-Person		
		Selected	Not selected	Total
**Web**	Selected	26	2	28
	Not Selected	4	54	58
	Total	30	56	86

### Comparability of household characteristics

The 83 cases with both a web and in-person complete and at least partial match status yielded 186 matching person pairs for comparison. We first examine person identifiers since this would be the simplest way to match persons between the two modes. Both the web and in-person screener ask for a full name to assist in enumerating all household members.

Table 3 shows the distribution of the personal identifier type provided in each mode. Person name (both full name and first name only) turned out to be poor by itself for matching persons between modes–successful for only 59% of the matching person pairs because of the low rate it was provided in the in-person screener. The web mode performed better (94.6%) vs in-person mode (36.6%) for collecting a full personal identifier, largely due to the high reporting of a generic label (e.g., man/woman, son/daughter, husband/wife, etc.) rather than a name for the in-person mode.

**Table 3:** Distribution of personal identifier type by mode.

	Full name	First only	Initials only	Generic label	Refused
**In-person**	36.6%	22.6%	0.5%	36.0%	4.3%
	68	42	1	67	8
**Web**	94.6%	3.8%	0.5%	--	1.1%
	176	7	1	0	2

Respondent counts provided below percentages.

Other characteristics examined include gender, age, race and ethnicity; characteristics that are used for sampling to determine if a person will be selected for the interview and exam components of NHANES. Table 4 shows the proportions matching between the web and in-person modes for these characteristics.

**Table 4:** Matching status between web and in-person modes for select demographic characteristics.

	Gender	Age^a^	Race	Ethnicity^a^
**Match**	99.5%	96.2%	87.4%	92.9%
	185	176	160	170
**Missing (web)** ^**b**^	0.5%	1.1%	0.0%	2.2%
	1	2	0	4
**Discrepant**	--	2.7%	12.6%	4.9%
	--	5	23	9

^a^ Excludes three persons where race was not collected for persons with another reported primary residence in the in-person mode.

^b^ No characteristics were missing for the in-person mode.

Respondent counts provided below percentages.

Generally, discrepancies were rare. Reported gender nearly uniformly matched between modes with the only mismatch due to item-nonresponse of one household member in the web mode. Despite being collected with different question formats, age showed few mismatches (3%), and still a high overall match rate between the two modes (97%). The web mode collected household member age as a category of ranges with a total of 12 categories available (from 0–11 months to 80+ years). The in-person mode collected date of birth, or directly reported age if date of birth is refused or unknown. For making comparisons between the two modes, the in-person age was recoded to match categories used in the web mode. The few discrepancies were not large and were never more than one age range apart. For example, a discrepant household members’ age may have been reported as 39 for the in-person mode, but on the web was reported as 40–49. Discrepancies increased for ethnicity, but the match proportion remained above 90 percent.

Race demonstrated somewhat lower consistency in terms of matching between the two modes. While the proportion matching was still high at 87 percent, it was the lowest among all characteristics. Table 5 shows where discrepancies occurred, with nearly all (21 out of 23 persons) occurring between White and Other for reported race. There is no clear direction between the two modes suggesting that one category is more likely in either self-administered (web) or interviewer-administered (in-person) mode.

**Table 5:** Matching status between web and in-person modes for reported race^a^.

	In-person sample Race			
**Web sample** **Race**	White	Black	Other	Asian
White	126	0	8	0
Black	0	14	0	0
Other	13	0	12	0
Asian	1	0	1	8

^a^ Excludes three persons where race was not collected for persons with another reported primary residence in the in-person mode.

Highlighted cells (grey) represent discrepant cells.

### Household mismatches

The previous section provided detail on how consistently household member characteristics were reported when the same person was reported in the web and in-person modes. As shown in Table 1, there were a few instances where only part of the household matched between modes with additional persons identified in either the web or the in-person mode. This occurred for 11 total households, yielding 12 orphaned persons–persons that do not have a corresponding match in another mode. The household composition for each partially matching household was reviewed to determine if any patterns could be determined for each mode. The misreporting observed was infrequent and not entirely contained within one mode, with details shown in Table 6.

**Table 6:** Details of households with partial matching status.

Status	Persons identified in both modes	Web added	In-person added	Age groups of additional persons
Partial Match Web+	3	1		3–5
Partial Match Web+	2	1		20–29
Partial Match Web+	1	1		20–29
Partial Match In-Person+	1		2	30–39 and 0–1
Partial Match In-Person+	1		1	50–59
Partial Match In-Person+	2		1	20–29
Partial Match In-Person+	1		1	60–69
Partial Match In-Person+	1		1	60–69
Partial Match In-Person+	1		1	20–29
Partial Match In-Person+	1		1	60–69
Partial Match In-Person+	1		1	60–69

For DUs where additional persons were identified in the web mode, one additional person was identified in each of three different DUs. Of these three persons, one was a child and the other two were adults. For DUs that identified additional persons for the in-person mode, nine persons across eight DUs were identified. With the exception of one person (an infant), the additional persons identified were adults.

With few cases where additional persons were identified in each mode, identifying a clear pattern to explain the discrepancy between modes is difficult. When looking at households who reported additional unmatched household members on the web screener than were reported to the interviewer, two of the three screener respondents initially did not report one person listed on the web screener and included other previously unnamed household members. The other screener respondent reported for only themself. The missed persons were younger and there were no indications they were spouses or partners, but rather a child of one of the household members, an adult child, or some other relative. In determining household relationships, we made judgements by looking at ages, surnames, or the use of generic identifiers (e.g., husband, daughter) for those reported. Collecting relationships between household members occurs only if someone is selected from the DU and was not collected at all for the web mode. For the in-person mode, only two DUs that identified additional persons resulted in selecting a person.

Contrastingly, for seven of the eight DUs with additional persons identified in the in-person mode (not reported for the web mode), the web screener respondent reported themselves and no other household members. The missing persons omitted from the web tended to be older and were presumed spouses or partners of the respondent based on our assessment. Unlike the omissions that occurred for in-person mode, the omissions for web appeared to be more systematic. This misreporting appears deliberate as respondents are first asked to report the number of household members living with them (reminding them to include themselves), then (after listing household members) asked a series of four questions to determine if anyone was missed.

## Discussion

The results of this pilot test provide a novel contribution to the empirical literature for web data collection. While there is an abundance of literature on mode effects for web versus interviewer administered modes, little is known about the comparability of household compositional data between these modes. We found that screening households by a web mode identified generally the same persons that in-person screening would identify. The observed rate of 84 percent for perfect household matches was relatively high, indicating that we identify most of the same household members with a web tool as we would with in-person visits. A longer length of time between modes did not have a clear effect on whether the households matched.

We noted earlier that the purpose of web screening for NHANES was to identify households that have no selected members and therefore need no in-person follow-up. Because the proportion of screened households where someone is selected is relatively low, this could result in time and cost savings. Some error in the reporting of the household composition can be tolerated as long as it does not affect the selection status of household members. Households that have one or more members selected would be visited in-person, where information would be verified and corrected as needed. This has broad applications to studies that first screen for subpopulations, such as the presence of children, specific health conditions, or demographic characteristics.

Seven percent of the 86 households that responded to both modes disagreed on whether one or more persons would be selected in the household. False positive cases, where the web mode determined household members would be selected, but the in-person did not, can be tolerated as they would not affect the final sample. These would instead result in inefficient and unnecessary interview visits. On the other hand, false negative cases, where the web mode determined that no household members would be selected, but the in-person mode found that household members actually would be selected, would require boosting the sample to account for the reduced efficiency. The false negative rate observed is 5%, which would have only a small effect on the projected sample required.

Personal identifiers were provided in 99% of the web responses and 96% of the in-person responses. However, the web mode elicited more substantive identifiers with full names provided in 95% of the web responses, while in-person responses were distributed between full names (37%), generic labels (36%), and only initials (23%). This observation is interesting in the context of our pilot study, since the web screener would have been completed prior to the in-person visit–where personal identifiers have already been revealed to the study. This suggests that respondents consider the request for personal identifiers of household members to be a sensitive request.

Beyond household-level matching, we found that consistency in the report of person specific characteristics is high but does decrease as the observed specificity of that measure decreases. The web and in-person screening instruments rely on a single household informant. Characteristics such as gender may be generally observable, but others such as age, race, or ethnicity can be more difficult. This is reflected in our findings with near perfect consistency for gender, but increasing error for age, ethnicity and race. Errors for age were small and it is possible that small errors could occur even when the person is well-known to the respondent. Observed errors for ethnicity and race are potentially more problematic, especially for studies like NHANES, which oversamples some minority groups. However, consistency remained high: 93 percent for ethnicity and 87 percent for race. For race, discrepancies were primarily between categories of White and Other. Since NHANES samples these groups at the same rates, this does not affect the SP selection status, however this could be problematic in estimating outcomes for different groups if not corrected.

While household-level matching was high, it was not perfect and failure to fully enumerate all household members occurred in both modes; with greater error occurring in the web mode. We cannot rule out true change in household composition since the time between the web and in-person reports averaged about one month. However, given the pattern for reporting observed for the web mode, true change seems unlikely. As shown in Table 6, we consistently observed the household informant reporting for himself or herself and excluding others (like spouses) where household ties are expected to be strong.

The pattern of misreporting for the web mode reveals a form of under-reporting for self-administered surveys not previously observed. Especially given other questions and reminders for ensuring all household members are accounted for. For the NHANES study, we hypothesize this observed pattern is likely reluctance on the part of the household respondent to report for other adults without their knowledge. Additional research testing this hypothesis would be valuable.

There are limitations to our study. Response to the web screener was lower than anticipated. However, this may have occurred for a number of reasons. First, monetary incentives were not provided to DUs to encourage their participation in the web screener. Second, field period for contacting DUs was limited to six weeks before the PSU opened for in-person contacts. This limited the number of mailed contacts to three (initial letter, postcard reminder, and nonresponse letter). Finally, the low response may be the result of the strong emphasis on the upcoming in-person visit. Our invitation letter was required to provide selected DUs a choice of modes, counter to current research on choice [30]. The invitation letters stated that the household would still be visited in-person by a “health representative…to determine your eligibility to participate in NHANES.” This notification was more prominently displayed compared to the push to web text in the same letters. These limitations emphasized in-person participation over the web mode. As a result, only 96 households completed the web screener.

It is important to note that field interviewers were unable to complete in-person screeners with 10 DUs for which web screeners were submitted. These cases were reviewed to address concern that completing the web screener may cause an increase for in-person nonresponse. For most of these cases, interviewers were unable to make contact with the DU; six resulted in noncontact; two refused to complete the screener; and two were finalized as vacant. People have become adept at avoiding contact which may imply that the six noncontact cases are veiled refusals. This would suggest that up to eight percent of those completing the web screener did not want an in-person visit. Understanding the motivation and concerns of those households is critical if web screeners are to be used as a first step towards recruitment into a larger survey.
